# Consumers’ awareness, attitude and associated factors towards self-medication in Hail, Saudi Arabia

**DOI:** 10.1371/journal.pone.0232322

**Published:** 2020-04-28

**Authors:** Mukhtar Ansari, Abdulrahman Alanazi, Afrasim Moin

**Affiliations:** 1 Department of Clinical Pharmacy, College of Pharmacy, University of Hail, Hail, Saudi Arabia; 2 Department of Pharmaceutics, College of Pharmacy, University of Hail, Hail, Saudi Arabia; University Lyon 1 Faculty of Dental Medicine, FRANCE

## Abstract

**Objectives:**

To determine the factors motivating the consumers towards self-medication, the intended indications and the consumers’ perceptions about complications that may arise due to its use.

**Design:**

Cross-sectional community based prospective study

**Place and duration of study:**

Community pharmacies located at various locations of Hail, Saudi Arabia from January 2019 to March 2019.

**Methods:**

The subjects of this cross-sectional study were people visiting community pharmacies for self-medication. Data on 663 participants was collected through a validated questionnaire prepared on the basis of WHO guidelines for the regulatory assessment of medicinal products for use in self-medication. Two trained data collectors visited the randomly selected community pharmacies, approached and interviewed the consumers purchasing medicines without prescriptions. Data was entered in SPSS and analyzed using descriptive and inferential analyses (alpha level = 0.05).

**Results:**

Out of 663 respondents, 68.6% were university graduates; and 33.9% were healthcare professionals. Consumers preferred self-medication mainly for headache (85.8%), cold and sore throat (80.8%), cough (75.7%) and fever (71.8%) with the justification that these illnesses were minor (90.2%), time saving (82.2%), convenient (74.7%), quicker relief (66.1%), and economical (61.2%). Occupation was significantly associated with the reasons for preferring self-medication (p<0.001). Similarly, age, gender and education were also significantly associated with most of the reasons for opting self-medication. People were quite aware about harmful consequences of self-medication. Multivariate logistic regression analysis showed that the respondents with lower education (AOR = 2.404 [95% CI: 1.579–3.661]), non-healthcare professionals (AOR = 1.712 [95% CI: 1.143–2.565]) and higher monthly income (AOR = 0.376 [95% CI: 0.236–0.599]) preferred self-medication (p <0.001, p = 0.009, p <0.001) respectively.

**Conclusion:**

Self-medication was prevalent among young university graduate males for minor ailments mainly due to convenience and time saving. Despite people beliefs about the harmful consequences of self-medication, its use was omnipresent particularly among the respondents with lower education, non-healthcare professionals and people with higher monthly income.

## Introduction

Self-medication (SM) refers to the use of any medication without the prescription of a licensed healthcare practitioner [1]. The use of SM is pervasive and it may even be more common than the use of prescribed medications [2].

Irrational use of medicines is a global challenge to both developed and developing nations [3]. Although various factors contribute, self-medication is the major reason for the ridiculous use of medicines [4]. The World Health Organization emphasizes the role of self-care, and there are several benefits owing to responsible use of self-medication such as reduction in visits to clinics or hospitals, and economical however, it could lead to polypharmacy, drug interactions, adverse drug reactions, development of resistance, wastage of money, prolonged suffering and drug dependence due to its irresponsible use [5–7].

Self-medication has an important place in healthcare system and it plays vital role in the management of minor ailments. Professional healthcare consultation is expensive or not readily available to the public thus, SM becomes an obvious healthcare choice, and it is a routine practice in Saudi Arabia [8, 9]. Like in other countries, the private sector pharmacies are the most accessible healthcare facilities in Saudi Arabia and people can easily purchase most of the medicines without prescription. In Arab countries including Saudi Arabia, the prevalence of SM ranges from 35% to 92% [10–14]. Literature shows that most of the studies related to self-medication have been conducted among healthcare professionals or students, and there are only few studies concerned with general public of Saudi Arabia [11, 15–17]. Self-medication has not got the attention it deserves as a research topic. Furthermore, this study is probably first of its kind in Hail.

Community pharmacies are the most accessible healthcare facilities in Saudi Arabia, and people can easily get their medications from there without the need of a valid prescription. This results in widespread irresponsible use of self-medication in the Kingdom [18]. Thus, keeping in mind the self-medication safety concerns among Saudi population, the proposed study was conceived with the objectives to determine the factors motivating the consumers towards SM, the intended indications and the consumers’ perceptions about complications that may arise due to its use.

The findings of the study will recommend the government or policy maker to implement strategies to increase the level of awareness among the public and to facilitate responsible use of self-medications in the country.

## Materials and methods

### Design and setting

This was a cross-sectional community based study conducted in community pharmacies situated at various locations of Hail, Saudi Arabia from January 2019 to March 2019. Hail is the capital of Hail Region and it lies in north-western Saudi Arabia.

### Study population, sample size and sampling procedure

General public, both male and female with age of 15 years and above, and willing to voluntarily participate were included in this study. Based on the population size of 510447 for people of age group 15 to 74 years, confidence level of 99%, margin of error 5%, and response distribution of 50%, the minimum effective sample size calculated for this study was 663 [19]. The sample size for this study was calculated by using Raosoft sample size calculator [20].

$$n=Z^{2}\;P\;\left(1-P\right)/d^{2}$$

Where, n = required sample size, P = disease prevalence Z = confidence level, d = margin of error.

To sample the above subjects, community pharmacies were considered as the appropriate place to find the study participants who visit the pharmacies for SM. We found twenty five pharmacies in Hail [21]. Every alternate community pharmacy from the above list was randomly selected. Every third individual, meeting the criteria, and visiting these pharmacies between 9 am to 12 pm and 4 pm to 10 pm were selected using systematic random sampling method.

### Instrument

A pre-tested questionnaire prepared on the basis of WHO guidelines for the regulatory assessment of medicinal products for use in self-medication was used as the instrument to collect the data [22]. The questionnaire was pretested among 67 subjects who were not the part of main study. The questionnaire composed of four sections. Section one (consumer’s demographic variables such as age, gender, education, occupation, nationality and monthly income), section two (the factors attracting consumers toward SM practices), section three (indications for which consumers preferred SM, and section four (consumers’ perceptions about complications that may arise due to SM).

Based on the fluency of language in the community, the researchers decided to use survey tool in the Arabic Language. Initially the questionnaire was in English and latter it was translated in the Arabic language by an expert translator and it was again retranslated in English to validate the questionnaire. Two data collectors received a prior training on data collection method before visiting the pharmacies, and approached the consumers buying medicines without prescriptions. After briefing about the purpose and significance of the study, they were enrolled, interviewed and the responses were recorded. The modality of data collection in the pharmacies was through interviewer- administered questionnaire.

Face and content validity of the questionnaire was performed by the research team and the expert from the college and through piloting the questionnaire among few consumers. The reliability of the questionnaire was determined through Cronbach’s alpha [23]. The analyses of the questionnaire found a Cronbach’s alpha value of 0.79. Additionally, the instrument was validated through factor analysis using Bartlett’s test of spherecity (p<0.001) and Kaiser Meyer Olkin measure of sampling adequacy (KMO) (0.54).

### Ethical considerations

The study proposal was reviewed for any ethical issues by Research Ethics Committee, University of Hail, Saudi Arabia. The consumers were convinced about the confidentiality of the data, and their written consents were taken before conducting the interview. They were also clearly informed that they have the right to leave the interview any time without mentioning the reason to do so.

### Data management and analysis

Data was analyzed using IBM Statistical Package for Social Sciences (SPSS) Inc., Chicago, USA version 21.0. Chi-square test was used to see the association between self-medication and different groups (demographics). Logistic regression analyses were performed to identify the factors significantly associated with SM. A p value of <0.05 was considered statistically significant throughout the analyses.

## Results

A total of 663 study participants were involved in this study. Among them, 568 (85.7%) were Saudis, and 95 (14.3%) were non-Saudis. A significant fraction (83.4%) of the participants were from the age group of 15 to 35 years. More than one half (54.3%) of the respondents represented university students and healthcare professionals (e.g. pharmacists, doctors, nurses, paramedical staffs etc.) with educational attainment of university graduate level (68.6%). Similarly, more than half (54.3%) of the participants had monthly income of less than 5,000 Saudi Riyals (Approximately 1333 USD) [Table 1].

**Table 1:** Demographic variables of the respondents (n = 663).

Variables		Number	Percentage
Gender	Male	395	59.6
	Female	268	40.4
Age	15–25	356	53.7
	26–35	197	29.7
	36–45	63	9.5
	46–55	47	7.1
Education	Diploma	50	7.5
	High School	108	16.3
	University graduate	455	68.6
	Post graduate	24	3.6
	Others (e.g. elementary school, intermediate etc.)	26	3.9
Occupation	Teaching	46	6.9
	Private sector employee	23	3.5
	Health professionals (e.g. pharmacists, doctors, nurses, paramedicals etc.)	225	33.9
	Student	135	20.4
	Administrative employee	78	11.8
	Business	23	3.5
	Housewife	94	14.2
	Others	39	5.9
Monthly income (Saudi Riyals) (1 USD = 3.75 SAR)	< 5000	360	54.3
	5000 to <10000	124	18.7
	10000 to <15000	32	4.8
	15000 to <20000	118	17.8
	20000 & Above	29	4.4

Majority of the respondents stated that they preferred SM as illness was minor (n = 598; 90.2%), avoidance of long waiting at clinics (n = 545; 82.2%), convenience (n = 495; 74.7%), quicker relief (n = 438; 66.1%), cheaper (n = 406; 61.2%), greater choice of treatment (n = 338; 51%), fewer crowds at pharmacy (n = 310; 46.8%), and influence of friends and relatives (n = 288; 43.4%) [Table 2].

**Table 2:** Reasons for preferring SM (n = 663).

Reasons	Yes, n (%)	No, n (%)	Don’t know, n (%)
Illness minor (not serious)	598 (90.2)	49 (7.4)	16 (2.4)
Avoidance of long waiting at clinics (time saving)	545 (82.2)	105 (15.8)	13 (2)
Convenience	495 (74.7)	117 (17.6)	51 (7.7)
Quicker relief	438 (66.1)	158 (23.8)	67 (10.1)
Economical (cheaper)	406 (61.2)	257 (38.8)	0 (0)
Greater choice for treatment	338 (51)	259 (39.1)	66 (10)
Own active role in healthcare	327 (49.3)	251 (37.9)	85 (12.8)
Fewer crowds at pharmacy	310 (46.8)	246 (37.1)	107 (16.1)
Motivation (suggestion) from friends/relatives	288 (43.4)	338 (51)	37 (5.6)
Prescribed drugs are less effective	199 (30)	315 (47.5)	149 (22.5)
Influence of media/advertisement/internet	198 (29.9)	429 (64.7)	36 (5.4)
Embarrassed of discussing own problems/symptoms	191 (28.8)	420 (63.3)	52 (7.8)

Occupation was significantly associated with the reasons for preferring SM. Similarly, age, gender and education were also significantly associated with most of the reasons for opting SM [Table 3].

**Table 3:** Association between sociodemographic characteristics and reasons for preferring SM (n = 663).

Reasons	Age P value	Gender P value	Education P value	Occupation P value
Illness minor (not serious)	0.014*	0.766	<0.001*	<0.001*
Quicker relief	0.005*	0.034*	<0.001*	<0.001*
Convenient	0.195	0.088	<0.001*	<0.001*
Avoidance of long waiting at clinics (time saving)	<0.001*	0.852	0.617	<0.001*
Cheaper (economical)	<0.001*	0.108	0.053	<0.001*
Embarrassed of discussing own problems (symptoms)	<0.001*	0.006*	<0.001*	<0.001*
Motivation from friends/relatives	0.014*	0.004*	0.001*	<0.001*
Own active role in healthcare	<0.001*	0.218	<0.001*	<0.001*
Prescribed drugs are less effective	<0.001*	0.202	<0.001*	<0.001*
Influence of internet/advertisement	0.007*	<0.001*	<0.001*	<0.001*
Greater choice for treatment	<0.001*	0.015*	<0.001*	<0.001*
Fewer crowds at pharmacy	0.377	<0.001*	<0.001*	<0.001*

* Significant at *p* value <0.05

The major illnesses or health problems for which people preferred SM include headache, cold and sore throat, cough and fever [S1 Fig].

Only one-third (33.5%) of the consumers agreed that SM is a safe practice in Saudi Arabia. Likewise, people were quite aware about harmful consequences of SM. In general, people strongly believed that SM could cause numerous complications [Table 4].

**Table 4:** Attitude about complications of SM (n = 663).

Components	Agree N (%)	Neutral/ Undecided N (%)	Disagree N (%)
There is wastage of money if actual disease is not identified	583 (87.9)	69 (10.4)	11 (1.7)
SM leads to inadequate or excessive dosage	581 (87.6)	49 (7.4)	33 (5)
There is incorrect self-diagnosis with reference to SM	558 (84.2)	78 (11.8)	27 (4.1)
SM leads to incorrect choice of therapy	553 (83.4)	89 (13.4)	21 (3.2)
SM leads to risk of dependence and abuse	500 (75.4)	120 (18.1)	43 (6.5)
SM leads to premature stoppage of antibiotics therapy	486 (73.3)	106 (16.0)	71 (10.7)
SM results in use of drugs in self-limiting conditions	473 (71.3)	176 (26.5)	14 (2.1)
There is failure to recognize or report ADRs due to SM	426 (64.2)	195 (29.4)	42 (6.3)
SM is a safe practice in Saudi Arabia	222 (33.5)	146 (22)	295 (44.5)

On univariate analysis, the study participant’s age (p = 0.005), gender (p = 0.002), education (p<0.001), occupation (p = 0.033) and monthly income (p<0.001) were associated with self-medication. Hence, multivariate logistic regression analyses were used to see the factors affecting SM. The results show that the respondents with lower education, non-healthcare professionals and people with higher monthly income were more likely to prefer SM (p <0.001, p = 0.009, p <0.001) respectively. Other demographic characteristics including age and gender had non-significant correlation with opting to SM [Table 5].

**Table 5:** Univariate and multivariate logistic regression analyses on association of demographic factors with SM as safe practice (n = 663).

Variables	Category	Self-medication as safe practice		COR (95% CI)	AOR (95% CI)
		Yes n (%)	No n (%)		
Age (years)	≤35	199 (36.0%)	354 (64.0%)	2.097(1.250, 3.521)*	0.741 (0.419, 1.311)
	>35	23 (20.9%)	87 (79.1%)	1	1
Gender	Male	107 (27.1%)	288 (72.9%)	0.568 (0.400, 0.807)*	0.692 (0.476, 1.005)
	Female	115 (42.9%)	153 (57.1%)	1	1
Education	High school or lower college	78 (49.4%)	80 (50.6%)	2.319 (1.575, 3.414)*	2.404 (1.579, 3.661)*
	Bachelor or higher	144 (28.5%)	361 (71.5%)	1	1
Occupation	Non-healthcare professional	166 (37.9%)	272 (62.1%)	1.521 (1.034, 2.236)*	1.712 (1.143, 2.565) *
	Healthcare professional	56 (24.9%)	169 (75.1%)	1	1
Income (Saudi Riyals)	<10000	191 (39.5%)	293(60.5%)	2.921 (1.873, 4.555)*	0.376 (0.236, 0.599) *
	>10000	31 (17.3%)	148 (82.7%)	1	1

*Statistically significant associations with p ≤ 0.05

## Discussion

This study was carried out to determine the factors motivating consumers towards SM, the intended indications and the consumers’ perceptions about complications that may arise due to its use. In brief, the study findings indicated that the use of SM was common despite detectable awareness among the respondents.

The practice of self-medication (SM) is widespread including Saudi Arabia and the Middle East. Although, self-medication has many possible benefits [24, 25], it is associated with multiple risks too [26, 27]. Thus, it is worthwhile to assess people’s awareness and attitude about SM.

In this study, we found that young aged people (15 to 35 years) were more involved in practicing SM and this is consistent with the study findings in UAE [28]. The most prominent users of SM were the participants with university degrees which aligns with a Chinese study [29]. Healthcare professionals and students were the dominant users (54.3%) of SM and the percentage was even higher (78% to 96.6%) in the Gulf Region [30–32]. Healthcare professionals practiced SM more because of their medical knowledge. The higher percentage in latter studies may be interpreted as the focus mainly on the students and healthcare professionals. However, this study included mixed population. People having lower (<10000 SAR) monthly income stated that SM was not safer and worried about risk factors while they practiced SM more [29]. This ambivalent behavior may be attributed to their lack of affordability of medical consultations and expensive investigations [Tables 1 and 5].

The major justifications towards preferring SM include–nonseriousness of illness, avoidance of long waiting at clinics or hospitals, convenience, and quicker relief along with economical gain. Other reasons they stated include greater choice of treatment, own active role in healthcare, and less crowd at pharmacies. Additionally, motivation from friends and relatives, and the influence of the media or the internet encouraged them towards practicing SM. Similar results have been reported in other studies [8, 24, 33, 34].

Headache, respiratory problems and fever are among the common minor ailments people face in their day to day life. Thus, the above clinical conditions were the most common for which SM was sought. The finding is supported by other studies [2, 28, 35].

People’s perception was mixed regarding safety issues of SM. Only 33.5% of the respondents believed that practicing SM was safe which contradicts with an Indian study where 66.6% reported that SM was safe for them [36]. The higher percentage of positive attitude in latter study is preceded by overwhelming prevalence of SM.

The study highlighted major complications or risks of self-medication as wastage of money if actual disease is not identified, incorrect dosage, incorrect self-diagnosis, incorrect choice of therapy (e.g., use of antibiotics in self-limiting conditions), risk of dependence and abuse, premature stoppage of antibiotics therapy, and failure to recognize or report ADRs. The findings of a review study by Ruiz are in line with this [7]. Similarly, a systemic review of misuse of antibiotics in Saudi population shows a high (41%-92%) prevalence of misuse of antibiotics, particularly among the children [37]. Study conducted by Bin Nafisah et al in Saudi Arabia also adds to the misuse of antibiotics in 35.5% of viral illnesses [38]. This reflects excessive misuse of antibiotics in self-limiting conditions.

Regarding associated factors, the negative sign of standard regression coefficient (β) for age, gender, and monthly income indicates that participants with age ≤ 35 years, and males with lower monthly income were less in agreement compared to their counterparts towards SM as safe practice. Findings of an Ethiopian study also highlights that higher monthly income encourages SM [39]. Looking over the gender, females were more in favor of SM which is reflected in an Ethiopian study. This is probably due to gender specific preferences of females who tend to connect and share with each other more often than males which might have encouraged them towards SM [39].

Participants with high school or lower college level of education were about two and half times (AOR = 2.404 [95% CI: 1.579–3.661]) more in favor of using SM compared to those having bachelor or higher level of education. This can be linked with lower knowledge about the harmful consequences of SM among participants with lower level of education [40]. Similarly, the respondents not related to healthcare using SM were nearly twice as many as the respondents involved in healthcare occupations (AOR = 1.712 [95% CI: 1.143–2.565]). This may be attributed to the fact that HCPs possessed more knowledge about both desired and harmful consequences of SM, and thus chose the intensity and frequency of its use may also be proportional to their medical knowledge [41].

### Study recommendations

With our findings about the use of SM, we would like to draw the attention of regulatory authorities to employ suitable interventions towards raising the awareness among the people about safe and effective use of SM, and to implement stricter regulation on dispensing OTC and non-OTC medicines. We would also like to recommend for multicenter studies at the national level to detect the extent of practice of SM and accordingly design awareness campaign.

### Study limitations

This study addresses one of the most important issues of public health in the Kingdom. However, the study had certain limitations too. This study was carried out in only one city of the Kingdom. Additionally, other factors that might have affected respondents towards opting for SM were not included in the study tool, e.g. health behavior (smoking, drinking, chronic illness, and physical activity), the quality of health services (perceived quality of care), and self-perception biases etc.

## Conclusions

Self-medication plays an important role in healthcare. However, its irresponsible use could lead to numerous drug related complications. Self-medication was common particularly among young university graduate males. People preferred self-medication for headache, respiratory problems, and fever mainly due to convenience, time saving and non-seriousness of the illnesses. Most of the perceived reasons to opt for self-medication were considerably associated with occupation, education and age of the consumers. Despite perceptible beliefs among the respondents that self-medication can lead to various complications, its use was pervasive particularly among the respondents with a lower education, non-healthcare professionals and people with higher monthly income. Thus, the findings of the study will recommend the government or policy maker to implement strategies to increase the level of public awareness about the consequences of self-medication and to facilitate responsible use of self-medications in the country.

## Supporting information

S1 Fig Indications for self-medication. (TIF)

S1 File Questionnaire for self-medication (English). (DOC)

S2 File Questionnaire for self-medication (Arabic). (DOC)

S1 Data (SAV)
