# Supplementary figures and images for: Consumers’ awareness, attitude and associated factors towards self-medication in Hail, Saudi Arabia
Source: PLoS One. 2020 Apr 28;15(4):e0232322. doi: 10.1371/journal.pone.0232322 (PMC7188286; doi:10.1371/journal.pone.0232322)

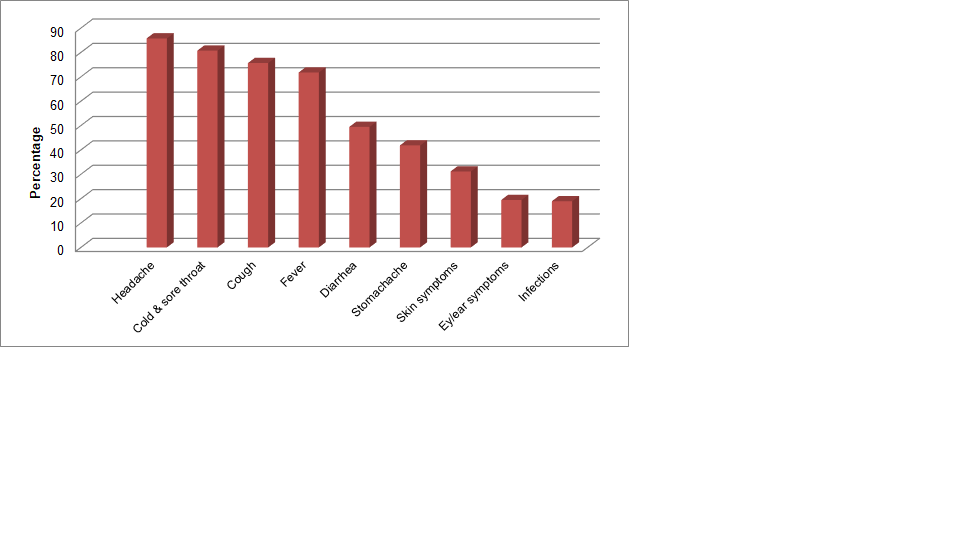

Supplement: S1 Fig — (TIF) [file pone.0232322.s001.tif]
