# Supplementary material for: Consumers’ awareness, attitude and associated factors towards self-medication in Hail, Saudi Arabia
Source: PLoS One. 2020 Apr 28;15(4):e0232322. doi: 10.1371/journal.pone.0232322 (PMC7188286; doi:10.1371/journal.pone.0232322)
Supplement: S1 File — (DOC) [file pone.0232322.s002.doc]

**Questionnaire for self-medication**

1. **Demographic characteristics of the consumers**

| **Age** | ………….years |
| --- | --- |
| **Gender** | 1🞏 Male  2🞏 Female |
| **Education** | 1🞏 No formal education  2🞏 Elementary school  3🞏 Intermediate school  4🞏 High School  5🞏 Diploma  6🞏 University graduate  7🞏 Post graduate  8🞏 Others (specify, if any):....................................... |
| **Occupation** | 1🞏 Housewife  2🞏 Administrative employee  3🞏 Business  4🞏 Nurse  5🞏 Pharmacist  6🞏 Doctor  7🞏 Paramedical staff  8🞏 Other (specify, if any):......................................... |
| **Nationality** | 1🞏 Saudi  2🞏 Non-Saudi |
| **Monthly income** | SAR……….. |

**B. Reasons for preferring self-medication** (*Please √ mark the* ***appropriate box***)

| **Items** | **1. Yes** | **2. No** | **3. Don’t know** |
| --- | --- | --- | --- |
| 1. Illness was minor/not serious |  |  |  |
| 1. Quick relief |  |  |  |
| 1. Convenient |  |  |  |
| 1. Time saving/avoidance of long waiting at clinic |  |  |  |
| 1. Cheaper |  |  |  |
| 1. Embarrassed of discussing own symptoms |  |  |  |
| 1. Motivation/suggestion from friends/relatives |  |  |  |
| 1. An active role in my own health care |  |  |  |
| 1. Physician’s prescribed drugs do not work well |  |  |  |
| 1. Influence of mass media/advertisement/internet |  |  |  |
| 1. Greater choice of treatment |  |  |  |
| 1. Fewer crowds at pharmacy |  |  |  |
| 1. Others (if any, please specify):……………………………………………… | | | |

**C. Indications for self-medication** (*Please √ mark the* ***appropriate box***)

| **Problems/illness** | **1. Yes** | **2. No** |
| --- | --- | --- |
| 1. Headache |  |  |
| 1. Cough |  |  |
| 1. Cold and sore throat |  |  |
| 1. Stomachache |  |  |
| 1. Fever |  |  |
| 1. Diarrhea |  |  |
| 1. Eye/ear symptoms |  |  |
| 1. Skin symptoms |  |  |
| 1. Infections |  |  |
| 1. Others (if any, please specify) :……………………………………………………… | | |

**D. Attitude about complications due to self-medications** (*Please* ***√*** *mark the appropriate level of your agreement or disagreement)*

| **Items/Components** | **Strongly**  **Agree** | **Agree** | **Neutral/**  **Undecided** | **Disagree** | **Strongly disagree** |
| --- | --- | --- | --- | --- | --- |
| Do you think self-medication is a safe practice in Saudi Arabia? |  |  |  |  |  |
| There is failure to recognize or report adverse drug reactions due to self-medication. |  |  |  |  |  |
| There is risk of double medication (with two different brands of same drug) or harmful interaction. |  |  |  |  |  |
| Self-medication may lead to inadequate or excessive dosage.  . |  |  |  |  |  |
| Self-medication may cause use of drugs in self-limiting conditions. |  |  |  |  |  |
| Self-medication may lead to unnecessary under-use or prolonged use of drugs. |  |  |  |  |  |
| Premature stoppage of antibiotics therapy  . |  |  |  |  |  |
| Incorrect self-diagnosis (e.g. severe diseases remain unnoticed) |  |  |  |  |  |
| Incorrect choice of therapy |  |  |  |  |  |
| Failure to recognize or self-diagnose contraindications, interactions, warnings and precautions etc. |  |  |  |  |  |
| Risk of dependence and abuse |  |  |  |  |  |
| Wastage of money if actual disease is not identified |  |  |  |  |  |
