# Supplementary material for: Consumers’ awareness, attitude and associated factors towards self-medication in Hail, Saudi Arabia
Source: PLoS One. 2020 Apr 28;15(4):e0232322. doi: 10.1371/journal.pone.0232322 (PMC7188286; doi:10.1371/journal.pone.0232322)
Supplement: S2 File — (DOC) [file pone.0232322.s003.doc]

**استبيان العلاج الذاتي**

ا. الخصائص الديموغرافية للمستهلكين

| **العمر** | ………………………………..سنة |
| --- | --- |
| **الجنس** | 1🞏 ذكر  2🞏 انثى |
| **التعليم** | 1🞏 غير متعلم  2🞏 ابتدائي  3🞏 متوسط  4🞏 ثانوي  5🞏 دبلوم  6🞏بكالوريوس  7🞏 دراسات عليا  8🞏غير ذلك حدد :..................................... |
| **الوظيفة** | 1🞏 ربة منزل  2🞏 موظف اداري  3🞏 رجل اعمال  4🞏 تمريض  5🞏 صيدلي  6🞏 طبيب  7🞏 موظف قطاع طبي  8🞏 غير ذلك حدد :..................................... |
| **الجنسية** | 1🞏 سعودي  2🞏غير سعودي |
| **الدخل الشهري** | …………………..ريال |

***ب. اسباب تفضيل التطبيب الذاتي (يرجى √ علامة في الخانة المناسبة)***

| **البند** | **نعم** | **لا** | **لا اعلم** |
| --- | --- | --- | --- |
| 1. مرض بسيط / ليس خطير |  |  |  |
| 1. سريع الشفاء |  |  |  |
| 1. قريب في المتناول |  |  |  |
| 1. توفير للوقت وعدم انتظار اوقات طويلة في عيادات الاطباء |  |  |  |
| 1. اقل تكلفة |  |  |  |
| 1. اخجل من مناقشة الاعراض الخاصة |  |  |  |
| 1. الدافع / اقتراح من أصدقاء / أقارب |  |  |  |
| 1. طريقة فاعلة للرعاية الصحية الذاتية |  |  |  |
| 1. الادوية التي بصفها الأطباء غير فعالة |  |  |  |
| 1. تأثير وسائل الإعلام / الإعلان / الانترنت |  |  |  |
| 1. خيارات اكثر للعلاج |  |  |  |
| 1. عدد أقل من الحشود في الصيدلية |  |  |  |
| 1. غير ذلك ارجو التحديد :……………………………………………… | | | |

ج. مؤشرات التطبيب الذاتي (يرجى √ علامة في الخانة المناسبة)

| **المشكلة / المرض** | **نعم** | **لا** |
| --- | --- | --- |
| 1. الصداع |  |  |
| 1. السعال |  |  |
| 1. نزلات البرد والتهاب الحلق |  |  |
| 1. وجع المعدة |  |  |
| 1. الحرارة |  |  |
| 1. الإسهال |  |  |
| 1. مشالك العيون و الانف |  |  |
| 1. مشاكل الجلد |  |  |
| 1. العدوى |  |  |
| 1. غير ذلك ارجو التحديد :……………………………………………… | | |

***الموقف من المضاعفات بسبب استخدام الأدوية ذاتياً (الرجاء وضع العلامة √ في المستوى المناسب من الاتفاق أو الاختلاف)***

| **البند** | **اتفق بشدة** | اتفق | **لا اعلم** | غيرمتفق | **غيرمتفق بشدة** |
| --- | --- | --- | --- | --- | --- |
| هل تعتقد أن التطبيب الذاتي هو الممارسة الآمنة في المملكة العربية السعودية؟ |  |  |  |  |  |
| هناك فشل في التعرف أو الإبلاغ عن التفاعلات الدوائية الضارة بسبب العلاج الذاتي. |  |  |  |  |  |
| هناك خطر في استخدام ادوية مزدوجة (منتجين من ماركتين من نفس الدواء) أو تفاعل ضار. |  |  |  |  |  |
| التطبيب الذاتي قد يؤدي إلى جرعة غير كافية أو مفرطة. |  |  |  |  |  |
| قد يؤدي التطبيب الذاتي الى اخذ ادوية في الحالات التي تشفى ذاتياً. |  |  |  |  |  |
| قد يؤدي العلاج الذاتي إلى الاستخدام غير الضروري أو الاستخدام المطول للأدوية. |  |  |  |  |  |
| ايقاف المضادات الحيويه في وقت سابق لاوانه. |  |  |  |  |  |
| التشخيص الذاتي غير الصحيح (مثل وجود الأمراض الحادة دون أن يلاحظها أحد) |  |  |  |  |  |
| اختيار غير صحيح للعلاج |  |  |  |  |  |
| الفشل في التعرف على موانع الاستعمال والتفاعلات والتحذيرات والاحتياطات وما إلى ذلك |  |  |  |  |  |
| خطر سوء الاستخدام و حصول الادمان |  |  |  |  |  |
| إهدار المال إذا لم يتم تحديد المرض الفعلي |  |  |  |  |  |
